# Functional Outcomes Associated With Blood Pressure Decrease After Endovascular Thrombectomy

**DOI:** 10.1001/jamanetworkopen.2024.6878

**Published:** 2024-04-17

**Authors:** Jae Wook Jung, Kwang Hyun Kim, Jaeseob Yun, Young Dae Kim, JoonNyung Heo, Hyungwoo Lee, Jin Kyo Choi, Il Hyung Lee, In Hwan Lim, Soon-Ho Hong, Byung Moon Kim, Dong Joon Kim, Na Young Shin, Bang-Hoon Cho, Seong Hwan Ahn, Hyungjong Park, Sung-Il Sohn, Jeong-Ho Hong, Tae-Jin Song, Yoonkyung Chang, Gyu Sik Kim, Kwon-Duk Seo, Kijeong Lee, Jun Young Chang, Jung Hwa Seo, Sukyoon Lee, Jang-Hyun Baek, Han-Jin Cho, Dong Hoon Shin, Jinkwon Kim, Joonsang Yoo, Minyoul Baik, Kyung-Yul Lee, Yo Han Jung, Yang-Ha Hwang, Chi Kyung Kim, Jae Guk Kim, Chan Joo Lee, Sungha Park, Soyoung Jeon, Hye Sun Lee, Sun U. Kwon, Oh Young Bang, Ji Hoe Heo, Hyo Suk Nam

**Affiliations:** 1 Department of Neurology, Yonsei University College of Medicine, Seoul, Korea; 2 Department of Radiology, Yonsei University College of Medicine, Seoul, Korea; 3 Department of Neurology, Korea University Anam Hospital and College of Medicine, Seoul, Korea; 4 Department of Neurology, Chosun University School of Medicine, Gwangju, Korea; 5 Department of Neurology, Brain Research Institute, Keimyung University School of Medicine, Daegu, Korea; 6 Department of Neurology, Seoul Hospital, Ewha Woman’s University, College of Medicine, Seoul, Korea; 7 Department of Neurology, Mokdong Hospital, Ewha Woman’s University College of Medicine, Seoul, Korea; 8 National Health Insurance Service, Ilsan Hospital, Goyang, Korea; 9 Department of Neurology, Asan Medical Center, University of Ulsan College of Medicine, Seoul, Korea; 10 Department of Neurology, Busan Paik Hospital, Inje University College of Medicine, Busan, South Korea; 11 Department of Neurology, Kangbuk Samsung Hospital, Sungkyunkwan University School of Medicine, Seoul, Korea; 12 Department of Neurology, Pusan National University School of Medicine, Busan, Korea; 13 Department of Neurology, Gachon University Gil Medical Center, Incheon, Korea; 14 Department of Neurology, Yongin Severance Hospital, Yonsei University College of Medicine, Yongin, Korea; 15 Department of Neurology, Gangnam Severance Hospital, Yonsei University College of Medicine, Seoul, Korea; 16 Department of Neurology, Kyungpook National University Hospital, School of Medicine, Kyungpook National University, Daegu, South Korea; 17 Department of Neurology, Korea University Guro Hospital and College of Medicine, Seoul, Korea; 18 Department of Neurology, Daejeon Eulji Medical Center, Eulji University School of Medicine, Daejon, Korea; 19 Department of Health Promotion, Severance Hospital, Yonsei University College of Medicine, Seoul, Republic of Korea; 20 Cardiovascular Research Institute, Yonsei University College of Medicine, Seoul, Republic of Korea; 21 Department of Research Affairs, Biostatistics Collaboration Unit, Yonsei University College of Medicine, Seoul, Korea; 22 Department of Neurology, Samsung Medical Center, Sungkyunkwan University School of Medicine, Seoul, Korea

## Abstract

**Importance:**

The associations between blood pressure (BP) decreases induced by medication and functional outcomes in patients with successful endovascular thrombectomy remain uncertain.

**Objective:**

To evaluate whether BP reductions induced by intravenous BP medications are associated with poor functional outcomes at 3 months.

**Design, Setting, and Participants:**

This cohort study was a post hoc analysis of the Outcome in Patients Treated With Intra-Arterial Thrombectomy–Optimal Blood Pressure Control trial, a comparison of intensive and conventional BP management during the 24 hours after successful recanalization from June 18, 2020, to November 28, 2022. This study included 302 patients who underwent endovascular thrombectomy, achieved successful recanalization, and exhibited elevated BP within 2 hours of successful recanalization at 19 stroke centers in South Korea.

**Exposure:**

A BP decrease was defined as at least 1 event of systolic BP less than 100 mm Hg. Patients were divided into medication-induced BP decrease (MIBD), spontaneous BP decrease (SpBD), and no BP decrease (NoBD) groups.

**Main Outcomes and Measures:**

The primary outcome was a modified Rankin scale score of 0 to 2 at 3 months, indicating functional independence. Primary safety outcomes were symptomatic intracerebral hemorrhage within 36 hours and mortality due to index stroke within 3 months.

**Results:**

Of the 302 patients (median [IQR] age, 75 [66-82] years; 180 [59.6%] men), 47 (15.6%)were in the MIBD group, 39 (12.9%) were in the SpBD group, and 216 (71.5%) were in the NoBD group. After adjustment for confounders, the MIBD group exhibited a significantly smaller proportion of patients with functional independence at 3 months compared with the NoBD group (adjusted odds ratio [AOR], 0.45; 95% CI, 0.20-0.98). There was no significant difference in functional independence between the SpBD and NoBD groups (AOR, 1.41; 95% CI, 0.58-3.49). Compared with the NoBD group, the MIBD group demonstrated higher odds of mortality within 3 months (AOR, 5.15; 95% CI, 1.42-19.4). The incidence of symptomatic intracerebral hemorrhage was not significantly different among the groups (MIBD vs NoBD: AOR, 1.89; 95% CI, 0.54-5.88; SpBD vs NoBD: AOR, 2.75; 95% CI, 0.76-9.46).

**Conclusions and Relevance:**

In this cohort study of patients with successful endovascular thrombectomy after stroke, MIBD within 24 hours after successful recanalization was associated with poor outcomes at 3 months. These findings suggested lowering systolic BP to below 100 mm Hg using BP medication might be harmful.

## Key Points

**Question:**

Is a medication-induced blood pressure (BP) decrease (systolic BP <100 mm Hg) during the 24 hours after successful endovascular thrombectomy associated with poor outcomes in patients with ischemic stroke?

**Findings:**

In a cohort study of 302 patients after successful endovascular thrombectomy, those experiencing medication-induced BP decreases exhibited a significantly lower odds of functional independence at 3 months (31.9%) compared with the no BP decrease group (49.1%), a significant difference. However, the odds of functional independence with spontaneous BP decrease did not significantly differ from those with no BP decrease.

**Meaning:**

The findings of this study suggest that a medication-induced BP decrease during the first 24 hours after successful reperfusion with endovascular thrombectomy may be harmful for patients with acute ischemic stroke.

## Teaser

This cohort study assesses whether use of medications to control blood pressure in patients who have undergone endovascular thrombectomy for acute ischemic stroke is associated with functional outcomes at 3 months.

## Introduction

Endovascular thrombectomy (EVT) is the standard of care in patients with acute stroke with large vessel occlusion.^1,2^ Along with efforts to expand indications for EVT, optimal control of blood pressure (BP) may further improve outcomes in patients with successful recanalization following EVT.^3^ Hypothetically, persistently elevated BP following EVT may increase the risk of intracerebral hemorrhage and cerebral edema,^4,5,6^ while excessively low BP may exacerbate ischemic injury due to decreased perfusion pressure in the vulnerable ischemic brain areas.^7,8^

Two recent randomized clinical trials have demonstrated that intensive BP management after successful EVT results in worse functional outcomes compared with conventional or less-intensive BP management.^9,10^ In the Outcome in Patients Treated With Intra-Arterial Thrombectomy–Optimal Blood Pressure Control (OPTIMAL-BP) trial, there was a sharp increase in the likelihood of poor outcomes as BP decreased in the intensive management group but not in the conventional management group. However, the exact reason for this disparity remains uncertain.

We hypothesized that the poor outcomes in the intensive BP management group were attributed to the excessive decrease in BP. Additionally, we postulated that outcomes may differ between patients with a medication-induced BP decrease (MIBD) and those with spontaneous BP decrease (SpBD). To test these hypotheses, we performed a secondary analysis of the OPTIMAL-BP trial.

## Methods

### Study Design and Population

This cohort study was a post hoc analysis of the OPTIMAL-BP trial, which compared intensive BP management (systolic BP [SBP] target <140 mm Hg) and conventional BP management (SBP 140-180 mm Hg) during the first 24 hours after successful reperfusion from June 18, 2020, to November 28, 2022, in patients who underwent EVT. Briefly, the OPTIMAL-BP trial was a multicenter, randomized, open-label trial with a blinded end point evaluation conducted across 19 stroke centers in South Korea. It included patients with acute ischemic stroke with large-vessel occlusion who achieved successful reperfusion of the occluded artery and exhibited an elevated SBP (≥140 mm Hg) within 2 hours of successful reperfusion.^10,11^ All patients in the primary analysis of the OPTIMAL-BP trial were eligible for this study. The study protocol was approved by the institutional review board of each participating hospital, and written informed consent was obtained from all participants or their approved surrogate. This study followed the Strengthening the Reporting of Observational Studies in Epidemiology (STROBE) reporting guideline for observational studies.^12^

### BP Monitoring and Management

Patient care was managed in a stroke unit or similar facility equipped with continuous BP monitoring, and BPs for all patients were recorded using noninvasive methods. Intravenous BP medications were used to achieve and maintain the target SBP of each group. Physicians primarily used nicardipine to reduce BP, although they could choose other drugs as needed. Data for the intravenous bolus or continuous infusion of BP medication were collected at 15-minute intervals. Data on the specific drug, infusion methods, dosage, duration, and initial administration time were recorded and analyzed using the individual BP medication record. In the conventional group, vasopressors were not used for SBP less than 140 mm Hg, and fluids or vasopressors were administered when hypotension required treatment at the physician’s judgment. The aim was to achieve the target SBP within 1 hour of randomization in both groups.

All sites provided time-stamped BP values within the first 24 hours after randomization. Blood pressure values were initially recorded at 15-minute intervals during the first hour, followed by hourly measurements for the subsequent 24 hours. After administering intravenous BP medication, BPs were measured at 15-minute intervals for the first hour, every 30 minutes for the following 2 hours, and then hourly for the remaining 24 hours.^11^ We did not impute any missing values.

### Definition of BP Decrease

The BP decrease was defined as at least 1 event of SBP less than 100 mm Hg during the 24 hours following randomization.^9,10^ To analyze hemodynamic variables associated with BP decrease, additional BP variables were determined using individual BP data: (1) number of BP decreases, (2) timing of the first BP decrease, (3) longest duration of sustained BP decrease, (4) accumulated time of BP decrease, and (5) duration from intravenous BP medication to BP decrease (eFigure 1 in Supplement 1).

### Study Group

Patients were categorized into 3 groups: the MIBD, SpBD, and the no BP (NoBD) groups. The MIBD group consisted of patients who experienced a BP decrease subsequent to administration of intravenous BP medication. Conversely, the SpBD group comprised patients who experienced a BP decrease without intravenous BP medication or before receiving intravenous BP medications. The NoBD group included patients who did not experience a BP decrease regardless of the use of intravenous BP medication.

### Outcomes

The primary outcome was a binary analysis of the modified Rankin Scale (mRS) score at 3 months, categorizing scores as either 0 (no symptoms) to 2 for functional independence or 3 for functional dependence to 6 (death). The primary safety outcomes involved symptomatic intracerebral hemorrhage within 36 hours and mortality associated with the index stroke within 3 months. The definition of symptomatic intracerebral hemorrhage was from the European Cooperative Acute Stroke Study III as any extravascular blood in the brain or within the cranium that was linked to clinical deterioration, defined by an increase of 4 points or more in the National Institutes of Health Stroke Scale score or death and identified as the main cause of the neurologic deterioration.^13^ Secondary outcomes included a shift analysis of the distribution of mRS scores, proportion of patients who achieved excellent recovery (a National Institutes of Health Stroke Scale score of 0-1 or an improvement of >8 points at 24 hours), successful reperfusion at 24 hours, and frequency of malignant cerebral edema within 36 hours. Successful reperfusion at 24 hours was defined as modified Thrombolysis in Cerebral Infarction grade of 2b, 2c, or 3 based on follow-up computed tomography angiography or magnetic resonance angiography at 24 (±12) hours. Malignant cerebral edema was defined as a rapidly worsening neurologic condition marked by significant brain swelling on computed tomography or magnetic resonance imaging, frequently resulting in death or adverse functional outcomes.^14^

### Statistical Analysis

Continuous variables are presented as a mean (SD) or median (IQR), and categorical variables are presented as number (percent). The Kruskal-Wallis, Wilcoxon rank-sums, χ^2^, or Fisher exact tests were used for comparing baseline characteristics as appropriate. To compare the BP variables of the MIBD and SpBD groups, all collected BP data points were linearly connected over time to generate an individual BP trend graph. Blood pressure variables were measured from individual trend graphs and compared by Wilcoxon rank-sum test. For the primary and secondary outcomes, binary logistic regression analyses were performed to calculate odds ratios (ORs) and 95% CIs and for the outcome of shift in mRS scores, the common OR was calculated using an ordinal logistic regression analysis. Adjusted ORs (AORs) were calculated using a multivariable logistic regression analysis adjusted for age, use of intravenous tissue-type plasminogen activator, National Institutes of Health Stroke Scale score immediately before EVT, and mean SBP over the first 24 hours. The Akaike information criterion was used to assess model fitting when constructing models. A 2-sided *P* < .05 value was considered statistically significant. All data were analyzed using R, version 4.2.2 (R Foundation for Statistical Computing) and PRISM, version 10 software (GraphPad PRISM Software).

## Results

Of the 306 patients who were enrolled in the OPTIMAL-BP trial, we analyzed data from 302 with available 3-month functional outcomes (eFigure 2 in Supplement 1). Among the 302 included patients, the median age was 75 (IQR, 66-82) years, 180 patients (59.6%) were men, 122 (40.4%) were women, BP decrease was observed in 86 (28.5%) patients, and 141 patients (46.7%) received intravenous BP medications. Patients were categorized into the MIBD (47 [15.6%]), SpBD (39 [12.9%]), and NoBD (216 [71.5%]) groups. Baseline characteristics did not differ substantially across the groups except SBP at enrollment and mean SBP for 24 hours, which were highest in the NoBD group, followed by the MIBD and SpBD groups. The proportion of patients receiving intensive BP management in the OPTIMAL-BP trial was highest in the MIBD group (93.6%) compared with the NoBD group (43.8%) and the SpBD group (38.5%). Within the SpBD group, 2 patients were administered intravenous BP medication following an elevation in BP that exceeded the targeted SBP (<180 mm Hg) after a BP decrease (Table 1).

**Table 1.** Baseline Characteristics According to Study Groups

Characteristic	Participants, No. (%)			
	MIBD (n = 47)	SpBD (n = 39)	NoBD (n = 216)	*P* value
Demographics and medical condition				
Age, median (IQR), y	78 (71-84)	78 (68-83)	74 (65-81)	.12
Sex				
Women	23 (48.9)	19 (48.7)	80 (37.0)	.17
Men, No. (%)	24 (51.1)	20 (51.3)	136 (63.0)	
Hypertension	33 (70.2)	27 (69.2)	171 (79.2)	.22
Diabetes	21 (44.7)	18 (46.2)	88 (40.7)	.76
Dyslipidemia	18 (38.3)	17 (43.6)	80 (37.0)	.74
Coronary artery occlusive disease	4 (8.5)	3 (7.7)	27 (12.5)	.71
Atrial fibrillation	22 (46.8)	22 (56.4)	102 (47.2)	.56
Congestive heart disease	4 (8.5)	2 (5.1)	8 (3.7)	.31
Previous stroke history	10 (21.3)	8 (20.5)	48 (22.2)	.97
Active cancer	3 (6.4)	1 (2.6)	10 (4.6)	.75
Smoking	10 (21.3)	7 (17.9)	51 (23.6)	.72
IV-tPA	12 (25.5)	14 (35.9)	72 (33.3)	.52
NIHSS score just before EVT >15	20 (42.6)	19 (48.7)	84 (38.9)	.50
Radiologic and procedural variables				
Site of occlusion in anterior circulation	41 (87.2)	34 (87.2)	197 (91.2)	.50
ASPECTS ≥6	42 (91.3)	37 (94.9)	205 (95.8)	.39
Good collateral	31 (70.5)	20 (52.6)	141 (69.5)	.11
mTICI score (immediate)				
2b	13 (27.7)	8 (20.5)	46 (21.3)	.50
2c	4 (8.5)	8 (20.5)	28 (13.0)	
3	30 (63.8)	23 (59.0)	142 (65.7)	
Time intervals				
Onset to puncture time, median (IQR), min	440 (303-707)	388 (230-484)	332 (206-730)	.21
Onset to enrollment time, median (IQR), min	540 (380-930)	480 (360-653)	450 (310-840)	.35
BP variables				
Previous antihypertensive treatment	23 (48.9)	18 (46.2)	112 (51.9)	.78
SBP at enrollment, median (IQR), mm Hg	150.0 (146.0-168.0)	146.0 (143.0-155.5)	152.0 (145.0-162.0)	.03
Mean SBP for 24 h, median (IQR), mm Hg	127.3 (122.5-133.5)	120.2 (114.3-125.2)	134.8 (130.1-141.9)	<.001
Intensive group of OPTIMAL-BP trial	44 (93.6)	15 (38.5)	96 (43.8)	<.001

Abbreviations: ASPECTS, Alberta Stroke Program Early CT Score; BP, blood pressure; EVT, endovascular thrombectomy; IV-tPA, intravenous tissue-type plasminogen activator; MIBD, medication-induced BP decrease; mTICI, modified Thrombolysis in Cerebral Infarction; NIHSS, National Institutes of Health Stroke Scale; NoBD, no BP decrease; OPTIMAL-BP, Outcome in Patients Treated With Intra-arterial Thrombectomy–BP Control; SBP, systolic BP; SpBD, spontaneous BP decrease.

### BP Measurements and Management

We evaluated 11 461 time-stamped BP recordings. A mean (SD) of 38.0 (20.4) BP recordings were performed per patient in the 24 hours following EVT (eFigure 3 in Supplement 1). Among the 141 patients given intravenous BP medication, 133 (94.3%) received nicardipine, 10 (7.0%) were administered labetalol, and 2 (1.4%) were treated with both drugs. The median total dosage was 20.0 (IQR, 5.0-49.4) mg for nicardipine and 3.8 (IQR, 1.3-8.4) mg for labetalol. The median time from enrollment to initial intravenous BP medication use was 0.8 (IQR, 0.0-2.0) hours, and the median duration of intravenous BP medication infusion was 4.0 (IQR, 1.3-7.5) hours. While the proportion of intravenous nicardipine use differed among the groups, no significant differences were observed in total dosage, timing of initial intravenous BP medication use, and duration of intravenous BP medication across the groups (Table 2).

**Table 2.** Variables of BP Management and Decrease

Variable^a^	Total	MIBD (n = 47)	SpBD (n = 39)	NoBD (n = 216)	*P* value
BP management (n = 302)					
Nicardipine, No. (%)	133 (44.0)	46 (97.9)	1 (2.6)	86 (39.8)	<.001
Labetalol	10 (3.3)	3 (6.4)	1 (2.6)	6 (2.8)	.39
Both	2 (0.7)	2 (4.3)	0 (0)	0 (0)	NA
Nicardipine dosage, median (IQR), mg	20.0 (5.0-49.4)	21.3 (11.0-54.1)	1.3 (1.3-1.3)	17.9 (4.5-45.8)	.15
Labetalol dosage, median (IQR), mg	3.8 (1.3-8.4)	2.5 (2.5-2.5)	7.5 (7.5-7.5)	3.1 (1.3-9.7)	.79
Total dosage, median (IQR), mg	20.0 (5.0-49.4)	21.3 (10.6-54.3)	1.3 (1.3-1.3)	17.9 (4.4-46.9)	.15
Interval between enrollment and initial administration of BP medication, median (IQR), h	0.8 (0.0-2.0)	0.5 (0.0-1.6)	9.5 (8.8-10.3)	0.8 (0.0-2.1)	.07
Duration of BP medication use, , median (IQR), h	4.0 (1.3-7.5)	4.8 (2.1-8.3)	0.9 (0.6-1.2)	3.5 (0.8-7.3)	.07
BP decrease (n = 86)					
No. of BP decreases, median (IQR)	1.5 (1.0-3.0)	1.0 (1.0-3.0)	2.0 (1.0-2.5)	NA	.59
Interval between enrollment and first BP decrease, median (IQR), h	5.6 (2.0-12.0)	6.3 (3.4-11.6)	4.0 (1.6-11.0)	NA	.20
Longest duration of sustained BP decrease, median (IQR), min	40.4 (12.3-78.0)	34.9 (11.5-53.9)	57.9 (18.1-131.6)	NA	.06
Cumulated time of BP decrease, median (IQR), min	50.7 (15.3-154.6)	34.9 (14.5-94.3)	82.3 (20.0-221.1)	NA	.07

Abbreviations: BP, blood pressure; MIBD, medication-induced BP decrease; NA, not applicable; NoBD, no BP decrease; SpBD, spontaneous BP decrease.

^a^ Statistical values of total dosages and duration were calculated in patients who received BP medication. For statistical analysis, the dose of labetalol divided by 10 was equated to the dose of nicardipine and then summed.

### BP Decrease

A BP decrease was observed in 86 patients (28.5%) and was more frequent in patients with vs without intravenous BP medication (49 [34.8%] vs 37 [23.0%] patients; *P* = .02). The BP decreases occurred at a median of 5.6 (IQR, 2.0-12.0) hours poststudy enrollment and occurred a median of 1.5 (IQR, 1.0-3.0) times within the first 24 hours. There were no significant differences between the MIBD and SpBD groups in the number, timing of the first, and duration of BP decreases (Table 2). In the MIBD group, the median time from intravenous BP medication administration to BP decrease was 0.25 (IQR, 0.25-0.75) hours (eFigure 4 in Supplement 1).

### Primary Outcome

In the unadjusted models, the proportion of patients who achieved functional independence at 3 months did not significantly differ between those with a BP decrease (either the MIBD or SpBD group) and those without a BP decrease (the NoBD group) (with, 40.7% vs without, 49.1%; crude OR, 0.71; 95% CI, 0.43-1.18; *P* = .19). However, a significantly lower proportion of patients in the MIBD group achieved functional independence (31.9%; OR, 0.49; 95% CI, 0.24-0.94; *P* = .03), unlike the SpBD group, which showed no significant difference (51.3%; OR, 1.09; 95% CI, 0.55-2.17; *P* = .80) compared with the NoBD group (Figure). On the multivariable analysis, the MIBD group exhibited a significantly smaller proportion of patients with functional independence at 3 months (AOR, 0.45; 95% CI, 0.20-0.98; *P* = .05), but the SpBD group did not show a significant difference (AOR, 1.41; 95% CI, 0.58-3.49; *P* = .46) compared with the NoBD group (Table 3).

**Figure.**
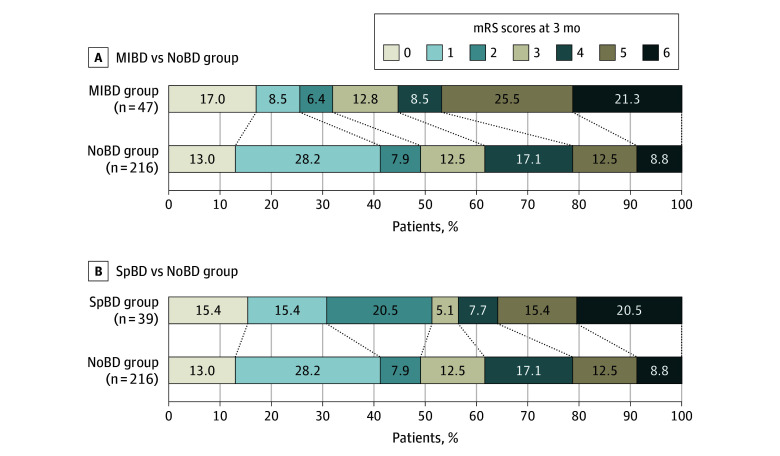
Distribution of Modified Rankin Scale (mRS) Score at 3 Months According to the Study Groups Comparison of mRS scores between the medication-induced blood pressure (BP) decrease (MIBD) group (A) and spontaneous BP decrease (SpBD) group (B) with the no BP decrease (NoBD) group. The median mRS score at 3 months was 4 (IQR, 1.5-5) in the MIBD group, 2 (IQR, 1-5) in the SpBD group, and 3 (IQR, 1-4) in the NoBD group. The mRS score ranges from 0 to 6, in which 0 denotes no symptoms and 6 represents death.

**Table 3.** Primary and Secondary Outcomes

Outcome	No./No. total (%)			MIBD vs NoBD, OR (95% CI)^a^		*P* value	SpBD vs NoBD, OR (95% CI)^a^		*P* value
	MIBD (n = 47)	SpBD (n = 39)	NoBD (n = 216)	Unadjusted	Adjusted		Unadjusted	Adjusted	
Primary outcome									
Functional independence at 3 mo (mRS score 0-2)	15/47 (31.9)	20/39 (51.3)	106/216 (49.1)	0.49 (0.24-0.94)	0.45 (0.20-0.98)	.05	1.09 (0.55-2.17)	1.41 (0.58-3.49)	.46
Primary safety outcomes									
Symptomatic intracerebral hemorrhage	5/47 (10.6)	6/39 (15.4)	15/216 (6.9)	1.60 (0.50-4.37)	1.89 (0.54-5.88)	.29	2.44 (0.82-6.47)	2.75 (0.76-9.46)	.11
Mortality associated with index stroke within 3 mo	7/47 (14.9)	3/39 (7.7)	10/216 (4.6)	3.60 (1.24-9.96)	5.15 (1.42-19.4)	.01	1.72 (0.37-5.94)	1.90 (0.34-9.04)	.43
Secondary outcomes									
Shift of mRS score (shift analysis)	NA	NA	NA	2.14 (1.20-3.84)	2.06 (1.11-3.85)	.02	1.42 (0.76-2.65)	1.25 (0.62-2.54)	.53
Excellent recovery in NIHSS score at 24 h	7/46 (15.2)	9/38 (23.7)	46/216 (21.3)	0.66 (0.26-1.50)	0.56 (0.21-1.34)	.22	1.15 (0.48-2.51)	0.76 (0.28-1.94)	.58
Successful reperfusion at 24 h	39/43 (90.7)	32/34 (94.1)	192/209 (91.9)	0.86 (0.30-3.12)	0.66 (0.21-2.52)	.50	1.42 (0.38-9.20)	0.88 (0.19-6.39)	.88
Malignant cerebral edema	4/47 (8.5)	4/39 (10.3)	6/216 (2.8)	3.26 (0.90-11.9)	4.22 (0.89-19.0)	.06	4.00 (0.98-14.7)	4.08 (0.73-22.0)	.10

Abbreviations: MIBD, medication-induced blood pressure decrease; mRS, modified Rankin Scale; NA, not applicable; NoBD, no blood pressure decrease; NIHSS, National Institutes of Health Stroke Scale; OR, odds ratio; SpBD, spontaneous blood pressure decrease.

^a^ Multivariable analysis of outcomes adjusted for variables of age, intravenous tissue-type plasminogen activator, NIHSS score before EVT, and mean systolic blood pressure over the first 24 hours.

### Primary Safety Outcomes

Twenty-six patients (8.6%) had symptomatic intracerebral hemorrhage and 20 patients (6.6%) died due to index stroke within 3 months. The incidence of symptomatic intracerebral hemorrhage was not significantly different among the groups (MIBD vs NoBD: AOR, 1.89; 95% CI, 0.54-5.88; *P* = .29; SpBD vs NoBD: AOR, 2.75; 95% CI, 0.76-9.46; *P* = .11). Compared with the NoBD group, index stroke-related mortality within 3 months was more frequent in the MIBD group (AOR, 5.15; 95% CI, 1.42-19.4; *P* = .01) but not in the SpBD group (AOR, 1.90; 95% CI, 0.34-9.04; *P* = .43) (Table 3).

### Secondary Outcomes

The mRS shift analysis indicated that the MIBD group exhibited significantly worse scores than the NoBD group (AOR, 2.06; 95% CI, 1.11-3.85; *P* = .02). However, the SpBD group did not show a significant difference in the mRS shift analysis. Compared with the NoBD group, neither the MIBD nor SpBD group demonstrated significant differences in proportions of excellent recovery at 24 hours, successful reperfusion at 24 hours, or malignant cerebral edema (Table 3).

## Discussion

In the OPTIMAL-BP trial population, episodes of SBP decreasing below 100 mm Hg were not infrequent, with a higher frequency in patients receiving intravenous BP medication. This study showed that the BP decrease was associated with worse functional outcomes and higher mortality at 3 months in the MIBD group, but not in the SpBD group compared with the NoBD group.

While the OPTMAL-BP trial enrolled patients with SBP higher than 140 mm Hg, episodes of SBP decreases below 100 mm Hg were observed in 28.5% of the patients. The frequency was similar to that in the Enhanced Control of Hypertension and Thrombectomy Stroke Study (ENCHANTED2/MT) (29.2%), which also enrolled patients with SBP higher than 140 mm Hg.^9^ In the present study, the BP decreased irrespective of the use of intravenous BP medication and assignment to the intensive or the conventional management group. However, BP decrease was more frequent in patients receiving intravenous BP medication or in the intensive group. We observed that a larger proportion of patients in the intensive management group received intravenous BP medications compared with those in the conventional management group, resulting in a greater frequency of BP decreases. The incidence of BP decrease was 38.1% in the intensive management group compared with 18.4% in the conventional group. A similar trend was observed in the ENCHANTED2/MT trial, where the incidence of BP decreases was 46% in the more intensive treatment group and 12% in the less intensive group. Results from both trials indicate an association between the use of intravenous BP medications for targeting a more intensive management of SBP goal and the occurrence of BP decrease.

Although episodes of BP decreases occurred after intravenous BP medication or were spontaneous, outcomes were poor when the BP decreased after intravenous BP medication. The MIBD group exhibited worse functional outcomes and higher mortality at 3 months compared with the NoBD group. However, outcomes were similar between the SpBD and NoBD groups. These findings partly explain why outcomes were worse in the intensive management group of the OPTIMAL-BP trial because intravenous BP medication was more frequently used in the intensive compared with the conventional management group. In this study, among patients with BP decreases, the episodes were infrequent and brief. In addition, the duration and number of BP decrease episodes did not significantly differ between the MIBD and SpBD groups over a 24-hour period. However, despite the comparable characteristics of BP decrease events, the MIBD group revealed worse outcomes. These findings suggest that an excessive BP decrease following intravenous BP medication may detrimentally affect the outcomes, even if it is brief or nonrepetitive.

The findings in this study suggest that the adverse effects of the BP decrease on outcomes may not be solely dependent on the magnitude of the BP decrease itself, but rather on the underlying cause of the decrease. Specifically, a BP decrease without the use of intravenous BP medication may reflect the resolution of a stressful situation, successful reperfusion after large vessel occlusion, or preserved autoregulation. Consequently, events of BP decrease in the SpBD group may not be associated with clinical deterioration. However, the BP decrease induced by intravenous BP medication in the MIBD group was associated with poor outcomes. Undesired hypotension on top of blunting the normal compensatory mechanisms may act as a secondary hit. In addition, ischemic brain areas are highly sensitive to perfusion pressure changes because of autoregulation failure.^15,16^ Blood pressure decrease due to intravenous BP medication can result in substantial hypoperfusion, which can be detrimental to the oligemic brain tissue. Cerebral autoregulation requires considerable time to be fully normalized. Studies have indicated that cerebral autoregulation impairments persisted until 24 to 72 hours, even after successful reperfusion.^17,18^ Our findings suggest that lowering BP using intravenous BP medication should be performed with caution at least for 24 hours after successful reperfusion. Further research in varied clinical settings is necessary to establish the external validity of these results.

### Limitations

This study has limitations. First, distinguishing the cause of BP decreases between the SpBD and MIBD groups was challenging. Blood pressure often naturally decreases after an acute ischemic stroke, especially among patients who have undergone successful reperfusion therapy.^19^ Second, the OPTIMAL-BP trial was conducted to regulate BP within 24 hours. The BP management and fluctuations beyond 24 hours may influence the functional outcome at 3 months. Third, the BP decrease was converted from a continuous to a dichotomous variable. The chosen threshold of SBP 100 mm Hg was derived from recent trials investigating lower SBP limits.^9,10^ Nevertheless, uncertainty exists regarding whether the criterion of SBP 100 mm Hg is appropriate for determining a clinically meaningful BP decrease. Furthermore, it is conceivable that the threshold affecting outcomes may vary among individual patients. In addition, the results of these post hoc analyses are considered exploratory, given the potential for type I errors due to multiple comparisons. Therefore, results should be interpreted with caution.

## Conclusions

In this cohort study, BP decreases induced by intravenous BP medication within 24 hours after successful EVT were associated with poor outcomes at 3 months. These findings suggest that lowering SBP below 100 mm Hg using intravenous BP medication may cause harm and underscore the importance of meticulous BP management. Our findings may also partly explain worse outcomes in the intensive management group of the OPTIMAL-BP trial.

## Notes

Supplement 1. **eFigure 1**. Individual Variation in SBP During 24 Hours After EVT **eFigure 2.** CONSORT Diagram **eFigure 3.** Mean Number of Hourly BP Measurements During Study Period **eFigure 4.** Patients’ Distribution of the MIBD Group According to the Time Interval From Administration of IV BP Medication to Initiation of BP Drop

Supplement 2.
Data Sharing Statement
