# Supplementary material for: Functional Outcomes Associated With Blood Pressure Decrease After Endovascular Thrombectomy
Source: JAMA Netw Open. 2024 Apr 17;7(4):e246878. doi: 10.1001/jamanetworkopen.2024.6878 (PMC11024769; doi:10.1001/jamanetworkopen.2024.6878)
Supplement: Supplement 1. — eFigure 1. Individual Variation in SBP During 24 Hours After EVT eFigure 2. CONSORT Diagram eFigure 3. Mean Number of Hourly BP Measurements During Study Period eFigure 4. Patients’ Distribution of the MIBD Group According to the Time Interval From Administration of IV BP Medication to Initiation of BP Drop [file jamanetwopen-e246878-s001.pdf]

Supplementary Online Content

Jung JW, Kim KH, Yun J, et al. Functional outcomes associated with blood pressure decrease after endovascular thrombectomy. *JAMA Netw Open*. 2024;7(4):e246878. doi:10.1001/jamanetworkopen.2024.6878

eFigure 1. Individual Variation in SBP During 24 Hours After EVT

eFigure 2. CONSORT Diagram

eFigure 3. Mean Number of Hourly BP Measurements During Study Period

eFigure 4. Patients' Distribution of the MIBD Group According to the Time Interval From Administration of IV BP Medication to Initiation of BP Drop

This supplementary material has been provided by the authors to give readers additional information about their work.

© 2024 Jung JW et al. *JAMA Network Open.*

eFigure 1. Individual variation in SBP during 24 hours after EVT

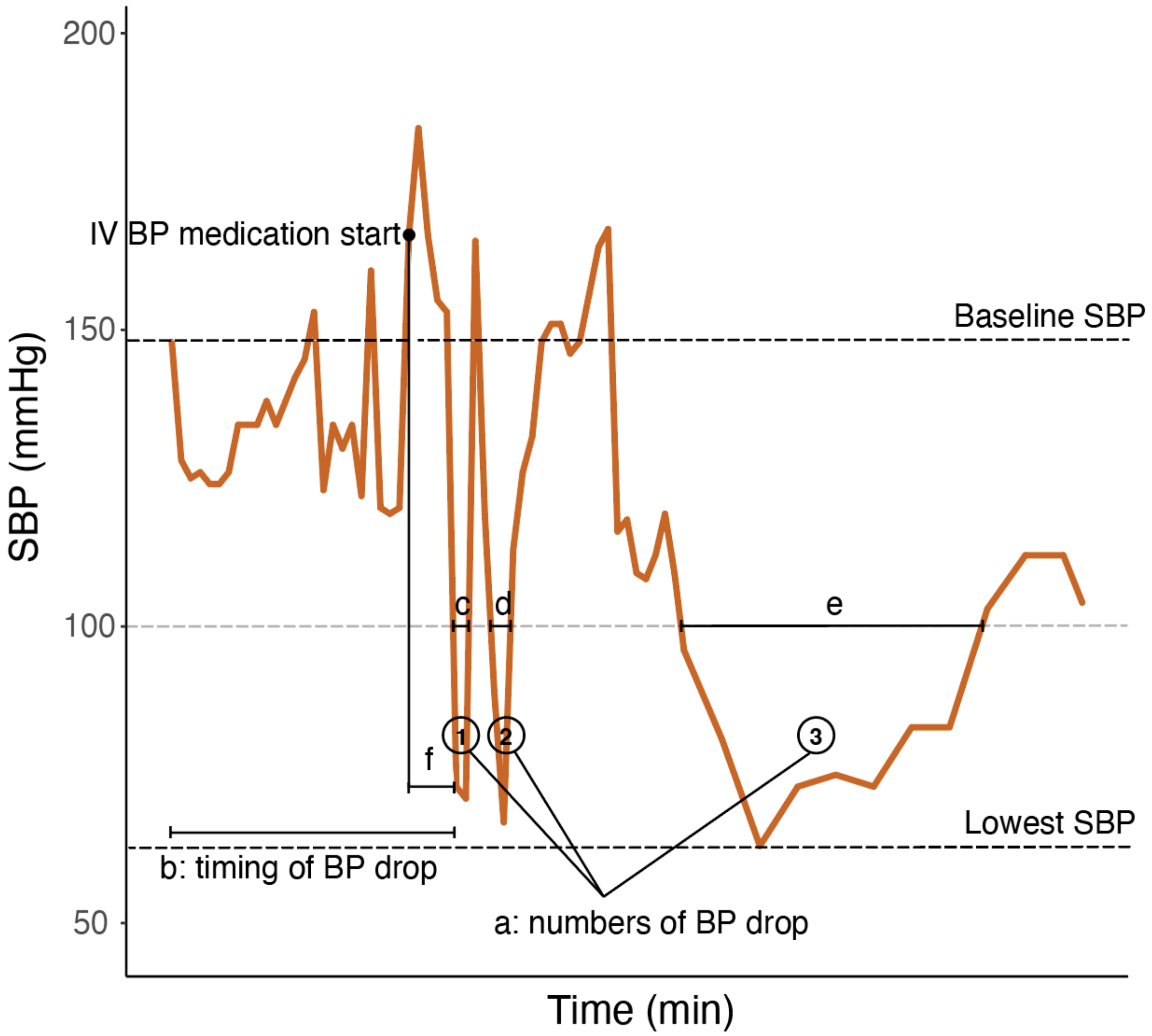

Abbreviations: BP, blood pressure; EVT, endovascular thrombectomy; IV, intravenous; SBP, systolic blood pressure.
Number of BP drop = a; timing of first BP drop = b; cumulated time of BP drop = c + d + e; longest continuous event of BP drop = e; duration from IV BP medication to BP drop = f

All collected BP readings were sequentially linked to construct a continuous blood pressure trend graph for each participant. Presented graph is the BP trend for a single participant in the OPTIMAL-BP trial.

© 2024 Jung JW et al. *JAMA Network Open.*

eFigure 2. CONSORT diagram

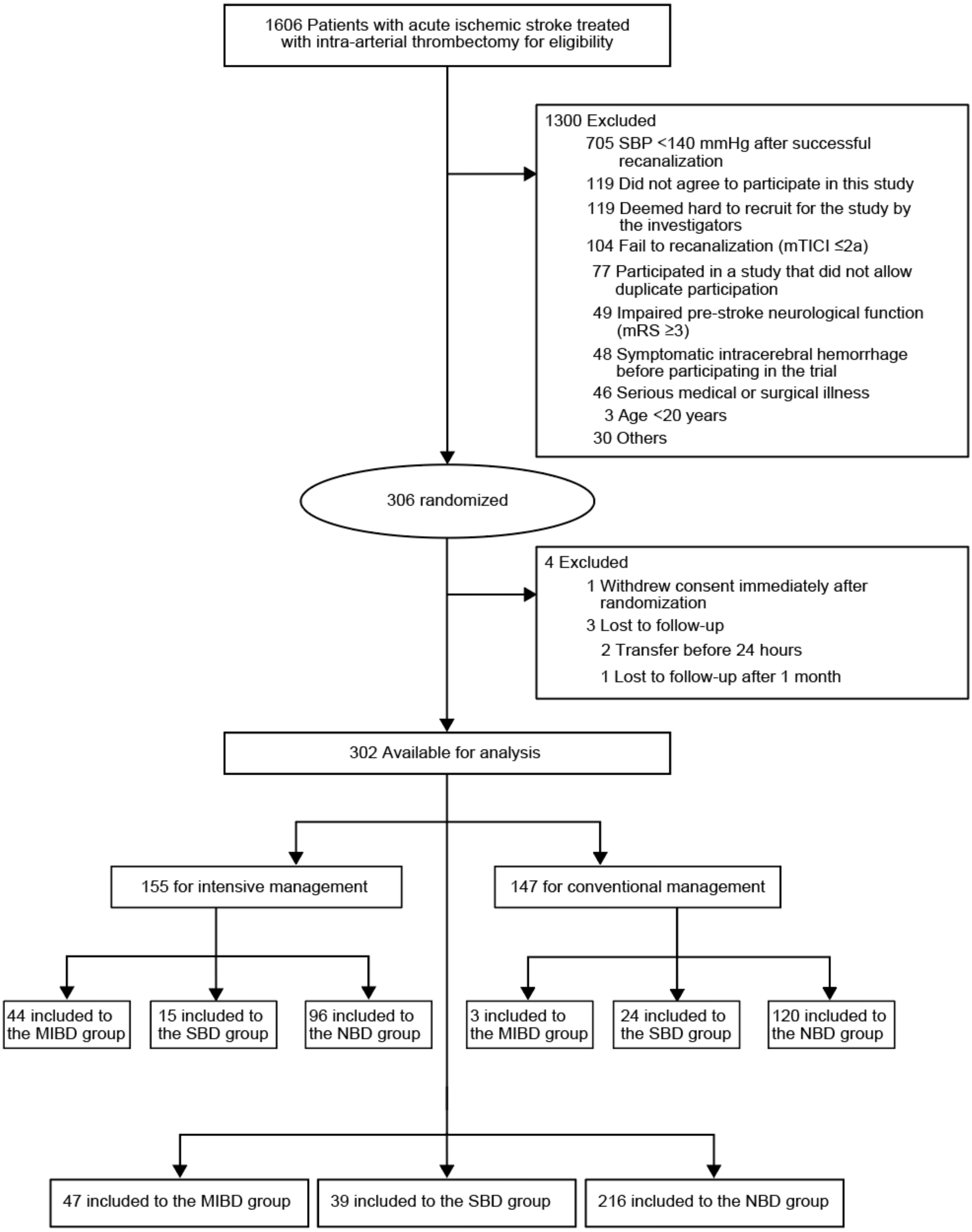

Abbreviations: MIBD, medication-induced blood pressure drop; mRS, modified Rankin Scale; NoBD, no blood pressure drop; SpBD, spontaneous blood pressure drop; mTICI, modified Thrombolysis in Cerebral Infarction.
306 participants were enrolled in the OPTIMAL-BP study. We excluded four patients who were removed from the parent study. Therefore 302 patients with sufficient data for analysis were included in this analysis.
[a]Fifteen patients were excluded for terminal cancer, 14 with cardiac or aortic disease. 4 with severre anemia and hematologic disease, 4 with chronic kidney disease, 4 with pneumonia, 2 with sepsis, 1 with cholecystitis, 1 with hemothorax, and 1 with radius fracture.
[b]Eleven patients were excluded for COVID-19 infection, 5 due to investigator error, 2 due to non-Korean citizenship, 2 due to immediate transfer to other hospitals, 2 due to no guardian, 2 for advanced dementia, and 6 for unknown reasons.

© 2024 Jung JW et al. *JAMA Network Open.*

eFigure 3. Mean Number of hourly BP measurements during study period

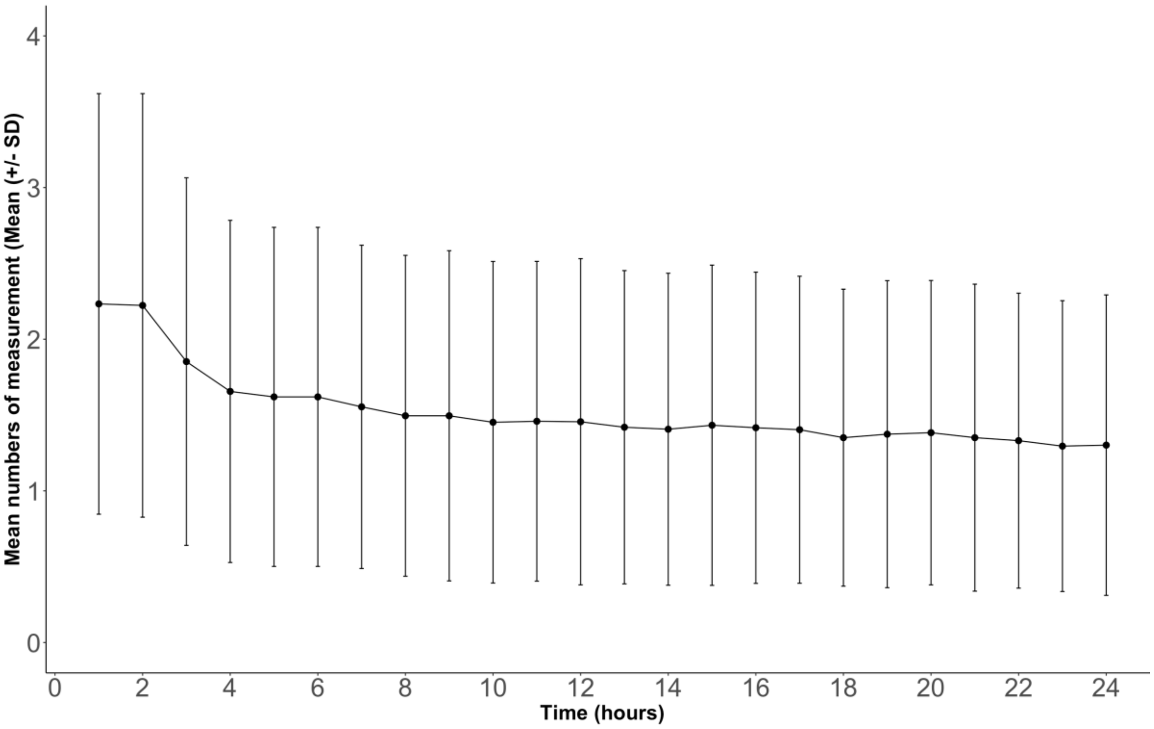

Abbreviations: BP, blood pressure.
Total of 11,461 time-stamped BP measurements of 302 patients. The mean number of recordings during 24-hour monitoring period was 38.0±20.4.

© 2024 Jung JW et al. *JAMA Network Open.*

eFigure 4. Patients' distribution of the MIBD group according to the time interval from administration of IV BP medication to initiation of BP drop

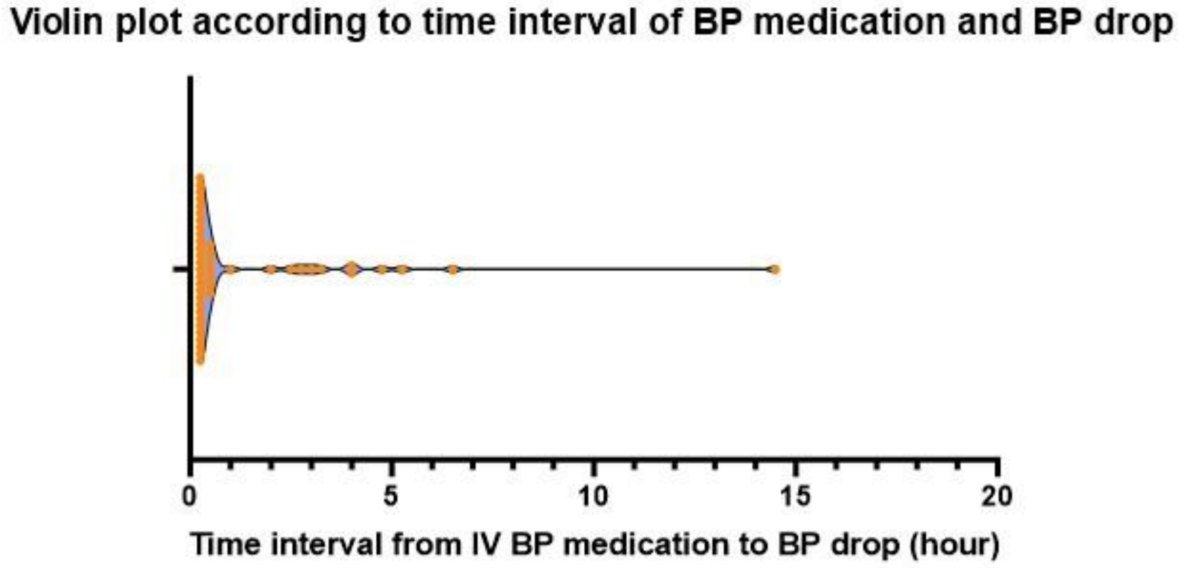

Abbreviations: BP, blood pressure; MIBD, medication-induced blood pressure drop; IV, intravenous.
Median time from administration of IV BP medication to BP drop was 0.25 (IQR, 0.25-0.75) hr.

© 2024 Jung JW et al. *JAMA Network Open.*
