# Supplementary material for: Functional Outcomes Associated With Blood Pressure Decrease After Endovascular Thrombectomy
Source: JAMA Netw Open. 2024 Apr 17;7(4):e246878. doi: 10.1001/jamanetworkopen.2024.6878 (PMC11024769; doi:10.1001/jamanetworkopen.2024.6878)
Supplement: Supplement 2. — Data Sharing Statement [file jamanetwopen-e246878-s002.pdf]

Data Sharing Statement

Jung. Functional Outcomes Associated With Blood Pressure Decrease After Endovascular Thrombectomy. *JAMA Netw Open*. Published April 17, 2024.
doi:10.1001/jamanetworkopen.2024.6878

Data
Data available: No
